# A PacBio Hi-Fi Genome Assembly of the Painter’s Mussel *Unio pictorum* (Linnaeus, 1758)

**DOI:** 10.1093/gbe/evad116

**Published:** 2023-06-21

**Authors:** André Gomes-dos-Santos, Elsa Froufe, André M Machado, Jasna Lajtner, Ján Černecký, L Filipe C. Castro, Manuel Lopes Lima

**Affiliations:** CIIMAR/CIMAR—Interdisciplinary Centre of Marine and Environmental Research, University of Porto, Matosinhos, Portugal; Department of Biology, Faculty of Sciences, University of Porto, Porto, Portugal; CIIMAR/CIMAR—Interdisciplinary Centre of Marine and Environmental Research, University of Porto, Matosinhos, Portugal; CIIMAR/CIMAR—Interdisciplinary Centre of Marine and Environmental Research, University of Porto, Matosinhos, Portugal; Department of Biology, Faculty of Sciences, University of Porto, Porto, Portugal; Department of Biology, Faculty of Science, University of Zagreb, Zagreb, Croatia; Institute of Landscape Ecology, Slovak Academy of Sciences, Nitra, Slovakia; CIIMAR/CIMAR—Interdisciplinary Centre of Marine and Environmental Research, University of Porto, Matosinhos, Portugal; Department of Biology, Faculty of Sciences, University of Porto, Porto, Portugal; BIOPOLIS Program in Genomics, Biodiversity and Ecosystems, CIBIO, Centro de Investigação em Biodiversidade e Recursos Genéticos, InBIO Laboratório Associado, Campus de Vairão, Universidade do Porto, Vairão, Portugal; IUCN SSC Mollusc Specialist Group, c/o IUCN, Cambridge, United Kingdom

**Keywords:** PacBio Hi-Fi, genome assembly, Unionida, freshwater mussels

## Abstract

The highly diverse group of freshwater mussels from order Unionida is found in the world’s freshwater systems due to several fascinating evolutionary adaptations, including “parental care,” and most notably, an obligatory parasitic phase in their early life cycle, called glochidia, which infests and uses fish for nutrition and dispersal. Freshwater mussels play essential ecological roles in freshwater habitats, including water filtration, sediment bioturbation, and nutrient cycling. However, these species are also highly threatened, being one of the faunal groups with the highest recorded extinction rate in the wild. Genomics methods have an incredible potential to promote biodiversity conservation, allowing the characterization of population health, identification of adaptive genetic elements, delineation of conservation units, and providing a framework for predictive assessments of the impact of anthropogenic threats and climate change. Unfortunately, only six freshwater mussel species have had their whole genomes sequenced to date, and only two of these are European species. Here, we present the first genome assembly of the Painter’s Mussel, *Unio pictorum* (Linnaeus, 1758), the type species representative of the order and the most widespread species of the genus in Europe. We used long-read PacBio Hi-Fi sequencing reads to produce a highly contiguous assembly that will pave the way for the study of European freshwater mussels in the Genome Era.

**Significance**

Freshwater mussels of the order Unionida are an inconspicuous but highly diverse group of strictly freshwater bivalves, with several fascinating biological and ecological features. Species in this group are declining worldwide; thus new ways of studying them are urgently needed to promote effective conservation measures. To date, only 6 freshwater mussel species (out of nearly 1,000) have had their whole genomes assembled. Here, we provide the first whole-genome assembly of the Painter’s Mussel *Unio pictorum* (Linnaeus, 1758). This high-quality genome assembly is a fundamental tool for studying many biological, ecological, and evolutionary features of this group of organisms, which will ultimately help to promote their conservation.

## Introduction

Unionida mussels are the most diverse group of strictly freshwater bivalves, comprising nearly 1,000 species in 6 families (Graf and Cummings 2021). Species in this group share several fascinating evolutionary traits that allow them to thrive in freshwater ecosystems. These include internal fertilization of eggs, often referred to as “parental care,” and most notably, their early life stage larvae (i.e., glochidia), which act as obligate parasites on freshwater fish (rarely other vertebrates) and use the hosts for food and river dispersal (Lopes-Lima et al. 2017a; Graf and Cummings 2021). Unionida are key organisms in freshwater habitats, playing essential roles such as water filtration, sediment bioturbation, oxygenation, and nutrient cycling (Vaughn et al. 2015; Lopes-Lima et al. 2017a; Graf and Cummings 2021). Although often inconspicuous to humans, the group has recently gained some general recognition due to the worrying records of global population declines (IPBES 2019; Lopes-Lima et al. 2021). Freshwater mussels are among the most threatened faunal groups, with an extinction rate in the wild of 5.9% (IPBES 2019; Lopes-Lima et al. 2021). There are several factors influencing their decline, that is, decrease in habitat quality, changes in hydrological regimes and conditions, the spread of invasive/alien species, and, more recently, droughts related to the climate crisis (Bogan 1993; Hastie et al. 2003; Nobles and Zhang 2011; Moore et al. 2019).

Applying genomics methods to the study of nonmodel organisms is fundamental for assessing biodiversity and promoting effective conservation (Allendorf et al. 2010; Meek and Larson 2019; Hohenlohe et al. 2021; Formenti et al. 2022). The whole-genome assembly (WGA) is arguably the most informative tool for a species biology, being among the most sought-after genomic resources for studying nonmodel organisms (Paez et al. 2022; Stephan et al. 2022; Theissinger et al. 2023). Despite this, the availability of WGA is still biased toward certain groups of the tree of life, with most invertebrates still being highly underrepresented (Hotaling et al. 2021). This is the case of freshwater mussels, with only six species having a reference genome assembly available (Renaut et al. 2018; Gomes-dos-Santos et al. 2021; Gomes-dos-Santos et al. 2023a; Gomes-dos-Santos et al. 2023b; Rogers et al. 2021; Smith 2021; Bai et al. 2022), of which only two are from European species: the freshwater pearl mussel *Margaritifera margaritifera* (Linnaeus, 1758) (Gomes-dos-Santos et al. 2021; Gomes-dos-Santos et al. 2023a) and the Iberian dolphin mussel, *Unio delphinus* Spengler 1793 (Gomes-dos-Santos et al. 2023b).

**Fig. 1:**
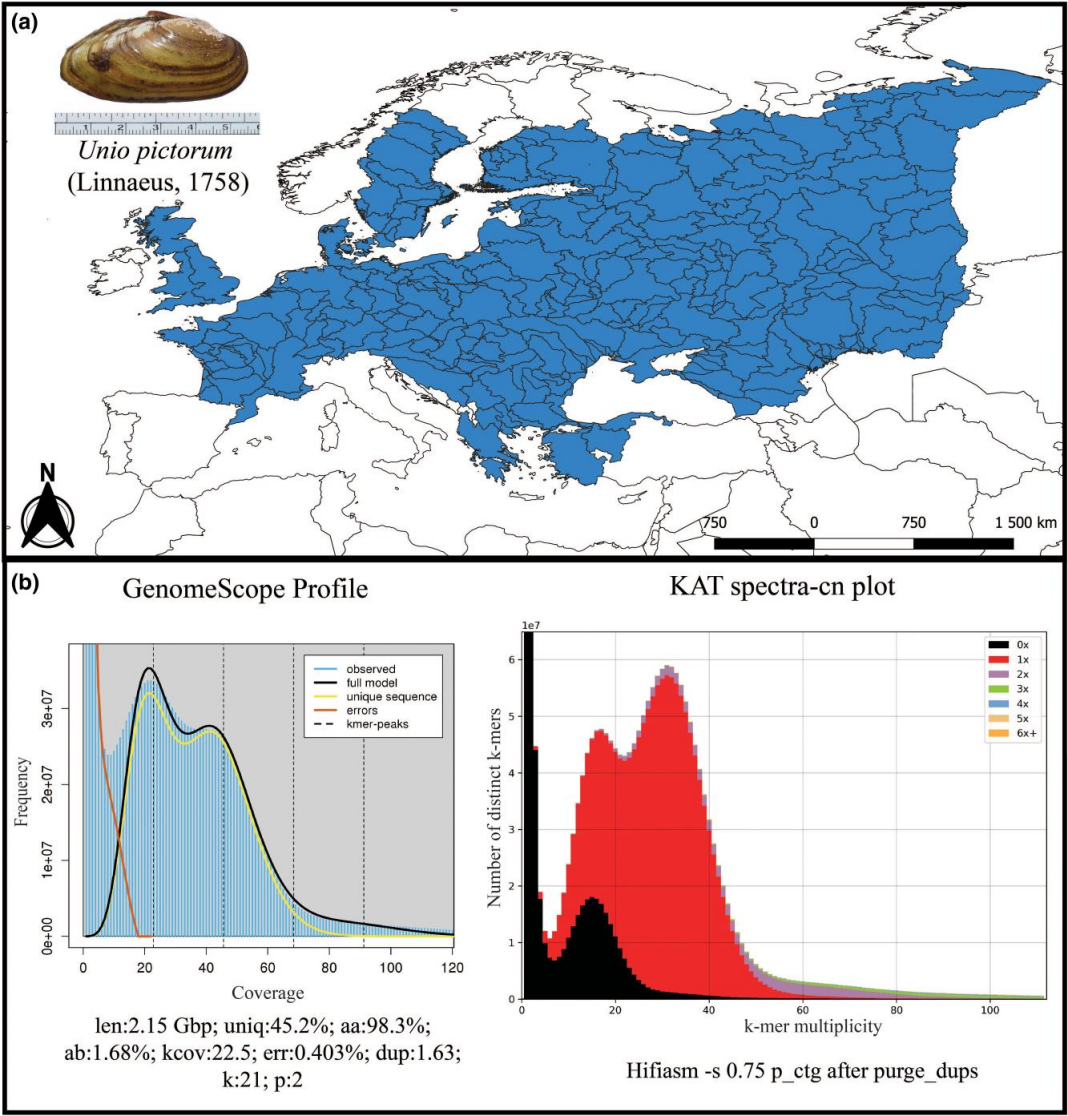
A) The map of the potential distribution of *Unio pictorum* generated by overlapping the points of recent presence records (obtained from 13) with level 5 polygons of the Hydrobasinslayer. On the top left is shown the *U. pictorum* specimen used for the WGA. B) Left: GenomeScope2 *k*-mer (21) distribution displaying the estimated genome size (len), homozygosity (aa), heterozygosity (ab), mean *k*-mer coverage for heterozygous bases (kcov), read error rate (err), the average rate of read duplications (dup), *k*-mer size used in the run (k:), and ploidy (p:). Right: Assessment of the *U. pictorum* genome assembly using the KAT comp tool to compare the PacBio Hi-Fi *k*-mer content within the genome assembly after running purge_dups. Different colors represent the read *k*-mer frequency in the assembly.

Here, we sequenced and assembled the first WGA of one of the most emblematic European freshwater mussel species, the Painter’s Mussel *Unio pictorum* (Linnaeus, 1758) (fig. 1*A*). The common name of this species derives from the fact that, historically, its shell was often used as a palette. The Painter’s Mussel is the type species of the whole order Unionida and one of the most widespread freshwater mussel species in Europe. The species is found from Great Britain to the Ural River in Russia, and is also present in Asia on the upper tributaries of the Ob River basin, in Russia and Kazakhstan (Lopes-Lima et al. 2017a; Vinarski et al. 2020; Babushkin et al. 2021; fig. 1*A*). The species shows considerable habitat plasticity, occurring in rivers, streams, flood plains, lakes, and even artificial freshwater habitats (e.g., reservoirs and fishponds; Lopez-Lima et al. 2017a). However, a worrying population decline has been recorded throughout its distribution in recent decades, with many local populations being considered threatened and several countries having very strict protection regulations for the species (Lopes-Lima et al. 2017a; Beran 2019). The genome produced here represents an important tool to explore in depth the many biological and evolutionary features of the Painter’s Mussel which will provide valuable guidelines to protect it, promote its conservation, and predict its adaptative potential in the face of future threats.

## Results and Discussion

### Genome Assembly

The pipeline used for the genome assembly is shown in detail in supplementary figure S1*A*, Supplementary Material online. Sequencing outputs resulted in a total of 22,881,671 PacBio Hi-Fi raw reads and 952,708,450 Illumina paired-end (PE) raw reads. GenomeScope2 estimated the genome size to be approximately 2.15 Gb and a duplication percentage of approximately 54.8% (fig. 1*B*). These estimates are similar to those reported for other recently available Unionida genomes (Renaut et al. 2018; Gomes-dos-Santos et al. 2021; Gomes-dos-Santos et al. 2023a; Gomes-dos-Santoset al. 2023b; Rogers et al. 2021; Smith 2021; Bai et al. 2022). GenomeScope2 estimated a relatively high percentage of heterozygosity, approximately 1.68% (fig. 1*B*), which has also been reported in the genomes of other Unioninae species (Rogers et al. 2021; Smith 2021; Gomes-dos-Santos et al. 2023b) and is common in molluscan genomes (Gomes-dos-Santos et al. 2020).

To account for the high levels of estimated heterozygosity, and following the approach recently applied to the only other genome available for the genus *Unio* (Gomes-dos-Santoset al. 2023b), several similarity thresholds for duplicate haplotypes to be purged (parameter -s) were tested for the Hifiasm genome assembly following the authors’ instructions (see Materials and Methods; supplementary table S1, Supplementary Material online). All values of the similarity threshold tested resulted in highly contiguous primary genome assemblies, all with <1,500 contigs and N50 lengths >9 Mb (supplementary table S1, Supplementary Material online). Lowering the thresholds resulted in a small decrease in the total number of contigs, with a small increase in the contiguity (supplementary table S1, Supplementary Material online). Given the small effect of each tested value on the contiguity of the assembly and the reduced and largely unchanged duplication values reported by the Benchmarking Universal Single-Copy Orthologs (BUSCO) analysis, the assembly generated using the default value (i.e., -s 0.75) was selected for further analysis and purged a posteriori using purge_dups. The resulting assembly showed that purge_dups was highly efficient in purging duplicated regions, reducing the number of contigs by more than half, increasing the contiguity, and having no effect on the overall BUSCO scores (table 1 and supplementary table S2, Supplementary Material online). The effectiveness of purge_dups in removing duplications is also observed in the *k*-mer frequency spectrum provided by K-mer analysis toolkit (KAT; fig. 1*B* and supplementary fig. S1*B*, Supplementary Material online), which shows low levels of duplicated *k*-mer (blue, green, purple, and orange in fig. 1*B* and supplementary fig. S1*B*, Supplementary Material online) and increased haplotype uniqueness (red in fig. 1*B* and supplementary fig. S1*B*, Supplementary Material online), similar to the *k*-mer distribution generated by GenomeScope2 (performed with Illumina PE reads, fig. 1*B*). Purge-dups reduced the content of diploid *k*-mers without significantly affecting the content of haploid *k*-mers (fig. 1*B* and supplementary fig. S1*B*, Supplementary Material online). Read back-mapping percentages, of short-read, RNA-seq, and long-read, were all above 94% (supplementary table S2, Supplementary Material online). The final purged genome assembly consisted of 670 contigs, with a total length of 2,434,378,075 bp, with a contig N50 of 10,612,599 and an L50 of 71 (table 1 and supplementary table S2, Supplementary Material online). The overall completeness of the genome assembly is also supported by the nearly 100% mapping rates of the PacBio Hi-Fi long reads used for the genome assembly, as well as Illumina whole genome and RNA-seq reads, both sequenced from a distinct individual from another population (supplementary table S2, Supplementary Material online).

**Table 1:** General Statistics of the *Unio pictorum* Final Genome Assembly (p_ctg); *U. pictorum* Alternative Haplotypes Genome Assemblies (hap1 and hap2); Other Published Freshwater Mussels Genome Assemblies

	Hifiasm -s 0.75 purge_dups p_ctg	Hifiasm -s 0.75 hap1	Hifiasm -s 0.75 hap2	*Megalonaias nervosa*	*Potamilus streckersoni*	*Margaritifera margaritifera* V1	*Margaritifera margaritifera* V2	*Unio delphinus*	*Venustaconcha ellipsiformis*	*Hyriopsis cumingii*
Total number of sequences	670	3,357	2,702	96,310	2,366	105,185	1,700	1,254	371,427	77.26
Total length (Gb)	2.43	2.44	2.35	2.36	1.77	2.47	2.45	2.50	1.59	3.38
N50 length (Mb)	10.61	3.59	3.66	0.050	2.05	0.29	3.43	10.91	0.006	84.3
L50	71	181	174	12,463	245	2,393	207	67	58,531	15
Largest contig (Mb)	44.85	26.90	20.62	0.58	10.78	2.51	23.80	43.58	0.31	158.3
GC content, %	34.82	34.84	2,698	35.82	33.79	35.42	35.30	35.07	34.19	36.07
Total BUSCO for the genome assembly (%)										
# Euk database	S:96.1% D:3.1% F:0.8%	S:92.5% D:2.7% F:2.0%	S:91.4% D:2.7% F:2.0%	S:70.2% D:0.4% F:14.9%	S:97.3% D:0.8% F:0.8%	S:85.8% D:1.0% F:5.9%	S:97.6% D:1.6% F:0.4%	S:96.1% D:2.4% F:1.6%	S:45.5% D:0.4% F:36.9%	S:92.2% D:0.8% F:3.1%
# Met database	S:93.7% D:2.6% F:2.4%	S:90.1% D:2.2% F:2.5%	S:90.6% D:2.3% F:2.8%	S:70.1% D:1.4% F:14.5%	S:93.6% D:1.4% F:2.3%	S:83.8% D:1.1% F:4.9%	S:95.5% D:1.4% F:2.0%	S:94.4% D:2.1% F:2.3%	S:52.8% D:0.9% F:29.7%	S:92.3% D:1.3% F:2.3%
Masking repetitive regions and gene prediction										
Percentage masked bases (%)	49.98	—	—	25.00	51.03	59.07	57.32	52.83	36.29	50.86
Number of mRNA	46,138	—	—	49,149	41,065	40,544	48,314	44,382	—	37,681
Protein coding genes (CDS)	46,138	—	—	49,149	41,065	35,119	48,314	44,382	—	37,681
Functional annotated genes	34,137	—	—	—	—	31,584	35,649	32,089	—	—
Total gene length (Gb)	0.83	—	—	—	—	0.90	1.13	0.86	—	—
Total BUSCO for the predicted proteins (%)										
+ Euk database	S:86.7% D:9.4% F:3.5%	—	—	—	—	S:81.2% D:9.4% F:3.9%	S:83.9% D:13.7% F:2.0%	S:88.2% D:8.6% F:2.7%	—	—
+ Met database	S:85.7% D:11.7% F:2.0%	—	—	—	—	S:82.3% D:10.3% F:3.2%	S:84.7% D:14.0% F:0.8%	S:86.0% D:11.3% F:2.3%	—	—

Note.—BUSCO scores are presented for the Eukaryota (Euk) and Metazoa (Met) databases, showing the percentages of Complete Single (S), Complete Duplicate (D) and Fragmented (F) hits. mRNA, messenger ribonucleic acid.

### Repetitive Elements Masking, Gene Model Predictions and Annotation

Genome masking covered almost half of the entire assembly, similar to the *U. delphinus* genome assembly and close to the initial GenomeScope estimate (fig. 1*B*, table 1). Similar percentages were observed for other Unionida genome assemblies (table 1). Overall, the occupancy of repetitive elements in the genome assembly was as follows: DNA elements with approximately 19.88% (ca. 484 Mb); unclassified with 15.16% (ca. 381 Mb); long interspersed nuclear elements (LINEs) with 7.96% (ca. 193 Mb); long terminal repeat elements (LTRs) with 3.93% (95.6 Mb); short interspersed nuclear elements (SINEs) with 2.54% (61.8 Mb); simple repeats with 0.43% (ca. 10 Mb); satellites with 0.17% (ca. 4.24 Mb); Small RNA with 0.02% (ca. 388 kb); and low complexity with >0.01% (56 kb). In contrast to other freshwater mussel genomes (Gomes-dos-Santos et al. 2021; Gomes-dos-Santos al. 2023a; Smith 2021; Bai et al. 2022), most repeats are classified as DNA elements rather than unclassified. Interestingly, DNA elements were also the most classified repeat type in the *U. delphinus* genome assembly (Gomes-dos-Santoset al. 2023b), which is the only other freshwater genome assembly obtained using PacBio Hi-Fi long reads. Although this is likely a synapomorphy, it may also be due to the high efficiency of the PacBio Hi-Fi reads in resolving repetitive regions, as the two *Unio* genome assemblies are by far the most contiguous freshwater mussel genome assemblies (table 1). Finally, a total of 46,138 protein-coding genes (CDS) were predicted by BRAKER2 (total length of 832,204,995 bp), of which 34,137 were functionally annotated by either InterProScan or BLAST searches (table 1). Both the number of gene predictions provided by BRAKER2 and the number of functionally annotated genes are well within the values recently reported for other freshwater mussel species (table 1) and within the average values observed in Mollusca (Gomes-dos-Santos et al. 2020). The overall quality of the gene prediction is also supported by the BUSCO scores obtained from the predicted protein, with almost no missing hits for any of the near-universal single-copy ortholog databases tested (table 1).

### Mitogenome Assembly

The mitogenome represents a highly valuable resource for phylogenetics and systematics studies of freshwater mussels (Lopes-Lima et al. 2017b; Froufe et al. 2019; Zieritz et al. 2021). However, the use of long-read approaches for these resources has not been thoroughly explored for this group (Gomes-dos-Santoset al. 2023a; Gomes-dos-Santoset al. 2023b). Here, we provide the assembly of the Painter’s Mussel mitogenome, using both a standard short-read assembly approach and a recently developed pipeline specifically designed for de novo assemblies using PacBio Hi-Fi long reads (Machado et al. 2022). The mitogenome assemblies are 15,756 bp (PacBio Hi-Fi reads) and 15,757 bp (PE short reads) long, consisting of 13 protein-coding genes, 22 transfer RNAs, and 2 ribosomal RNAs. Both assemblies were circularized and showed the same gene arrangement, as expected for female mitochondrial genomes of the subfamily Unionidae, commonly referred to as UF1 (Lopes-Lima et al. 2017b). Several of the PacBio Hi-Fi reads spawn the entire mitogenome, supporting the inferred structure of both assemblies. These results, together with the two recently assembled mitogenomes of *U. delphinus* and *M. margaritifera* using PacBio long reads (Gomes-dos-Santoset al. 2023a; Gomes-dos-Santoset al. 2023b), suggest that unlike other bivalves (Calcino et al. 2020; Formenti et al. 2021; Ghiselli et al. 2021), mitogenome assemblies using short-read approaches are still reliable for accurate compositional and structural assemblies.

### Conclusion

We provide the first genome assembly of the Painter's Mussel, one of the most widespread freshwater mussel species in Europe. The contiguity and completeness of the Painter's Mussel genome produced here are demonstrated by the use of multiple metrics. This assembly represents a key resource for this emblematic species, providing a critical tool to explore many of its ecological, biological, and evolutionary traits.

## Materials and Methods

### Sampling, DNA Extraction, Library Construction, and Sequencing

Two *U. pictorum* specimens were collected in the Dobra River (“45.515500, 15.473240,” Croatia, 2019, Voucher: BIV6631) and Danube River (“48.209152, 19.540361,” Slovakia in 2022, Voucher: BIV9798). Samples were transported to the laboratory, where tissues were separated, flash-frozen, and stored at −80 °C. Both shells and tissues are deposited in the CIIMAR tissue and mussel collection.

Genomic DNA extraction for Illumina short-read sequencing was performed with the Qiagen MagAttract HMW DNA extraction kit (Dobra), using foot tissue. Extracted DNA was sent to Macrogen Inc., for standard Illumina Truseq Nano DNA library preparation, followed by whole-genome sequencing of 150 bp PE reads, which was performed using an Illumina HiSeq X machine. Despite these attempts, the DNA extractions from this sample did not fulfill the minimum molecular weight requirements for PacBio sequencing. Consequently, the second individual (Danube) was collected, and DNA extraction was performed using the same methodology. The PacBio long-read Single Hi-Fi sequencing was conducted at Brigham Young University (BYU). Foot tissue was sent to BYU for high-molecular-weight DNA extraction followed by PacBio Hi-Fi library construction and sequencing, according to the manufacturer's recommendations (https://www.pacb.com/wp-content/uploads/Procedure-Checklist-Preparing-HiFi-SMRTbell-Libraries-using-SMRTbell-Express-Template-Prep-Kit-2.0.pdf). Size selection was achieved using the SageELF system. Sequencing was conducted on five single-molecule, real-time (SMRT) cells using the Sequel II system v.9.0, with 30 h run time and 2.9 h preextension. The circular consensus analysis was performed in SMRT® Link v9.0 (https://www.pacb.com/wp-content/uploads/SMRT_Link_Installation_v90.pdf) using default settings.

### Preassembly Processing

The general characteristics of the *U. pictorum* genome were estimated through a *k*-mer frequency spectrum analysis, using the PE reads (BIV6631). Raw sequencing PE reads were quality trimmed with Trimmomatic v.0.38 (Bolger et al. 2014), specifying the parameters “LEADING: 5 TRAILING: 5 SLIDINGWINDOW: 5:20 MINLEN: 36.” The quality of the raw and clean reads was validated in https://www.bioinformatics.babraham.ac.uk/projects/fastqc/ before and after trimming. Clean reads were used for genome size estimation using Jellyfish v.2.2.10 and GenomeScope2 (Ranallo-Benavidez et al. 2020), with a *k*-mer length of 21.

### Genome Assembly

PacBio Hi-Fi reads were assembled using Hifiasm 0.16.1-r375 (Cheng et al. 2021, 2022) testing a combination of multiple parameters, that is, *s* = 0.75, 0.55, 0.50, 0.45, 0.35, following the authors’ recommendations (https://hifiasm.readthedocs.io/en/latest/faq.html#p-large). The overall quality of these preliminary assemblies was accessed using the Quality Assessment Tool for Genome Assemblies (QUAST) v.5.0.2 (Gurevich et al. 2013) and the BUSCO v.5.2.2 (Manni et al. 2021) with Eukaryota and Metazoa databases. The assembly -s 0.75 was selected as the best assembly, and purge_dups v.1.2.5 (Guan et al. 2020) was used to further separate poorly resolved pseudo-haplotypes, specifying 23 as the transition between haploid and diploid cutoff and 5 and 96 as the lower and upper bounds for read depth, respectively. The cutoff values were determined by manual inspection of the *k*-mer frequency distribution plot produced by the KAT tool (Mapleson et al. 2017), and the resulting purged assemblies were evaluated using QUAST v.5.0.2 and BUSCO v.5.2.2, as described above. Assembly quality was accessed for completeness, heterozygosity, and collapse of repetitive regions using a *k*-mer distribution with KAT (Mapleson et al. 2017) and with read-back mapping, performed with PE using Burrows–Wheeler Aligner v.0.7.17-r1198 (Li 2013), for long reads with Minimap2 v.2.17, and for RNA-seq (SRR19261767; Gomes-dos-Santos et al. 2022) with HISAT2 v.2.2.0 (Kim et al. 2015).

### Repetitive Elements Masking, Gene Model Predictions and Annotation

RepeatModeler v.2.0.133 (Smit and Hubley 2015b) was first used to construct a de novo library of repeats of the *U. pictorum* genome assembly, which was subsequently used, along with the “Bivalvia” libraries from Dfam_consensus-20170127 and RepBase-20181026, for repetitive masking with RepeatMasker v.4.0.734 (Smit and Hubley 2015a).

Gene prediction was performed on the soft-masked genome assembly, using the BRAKER2 pipeline v2.1.6 (Brůna et al. 2021), using both RNA-Seq and protein spliced alignments as extrinsic evidence data. For the RNA-seq, the recently sequenced *U. pictorum* RNA-seq was retrieved from GenBank (SRR19261767; Gomes-dos-Santos et al. 2022), quality trimmed with Trimmomatic v.0.3839 (parameters described above) and aligned to the assembly using HISAT2 v.2.2.0 with the default parameters. For the protein data set, the complete proteome of 14 mollusc species and 3 reference Metazoa genomes (*Homo sapiens*, *Ciona intestinalis*, *Strongylocentrotus purpuratus*) was retrieved from public databases (following Gomes-dos-Santoset al. 2023b). BRAKER2 was applied using the parameters “–etpmode; –softmasking;” and after, AGAT v.0.8.0 (Dainat et al. 2020) was used for renaming, cleaning, and filtering gene predictions, as well as correcting overlapping predictions and removing coding sequence regions (CDS) with <100 amino acid and incomplete gene predictions (i.e., without start and/or stop codons).

Functional annotation was accomplished by applying both InterProScan v.5.44.80 (Quevillon et al. 2005) and BLASTP searches against the RefSeq database (Pruitt et al. 2007). DIAMOND v.2.0.11.149 (Buchfink et al. 2015) was used for homology searches, specifying the parameters “-k 1, -b 20, -e 1e-5, –sensitive, –outfmt 6.” Finally, BUSCO scores were accessed for the predicted proteins, as described above.

### Mitogenome Assembly

For PE reads, the mitogenome was obtained from the clean reads using GetOrganelle v1.7.1 (Jin et al. 2020). For PacBio Hi-Fi reads, a pipeline recently developed by the team was used (Machado et al. 2022). Mitogenome annotation was performed using MitoZ v.3.4 (Meng et al. 2019) with parameters (--genetic_code 5 --clade Mollusca), using the PE reads for coverage plotting.

## Supplementary Material

evad116_Supplementary_Data

## Data Availability

The raw read sequencing outputs were deposited at the NCBI Sequence Read Archive with the accession's numbers: SRR24657780-SRR24657789 (bam files) and SRR23693026-SRR23693030 (fastq files) for PacBio CCS Hi-Fi and respective subreads; SRR23693025 for Illumina PE. The Genome assembly is available under accession number JARLTB000000000. BioSample accession numbers are SAMN33562118 (sample BIV9798) and SAMN28495235 (sample BIV6631), and BioProject PRJNA940338. Mitochondrial genome assembly's accessions are OQ564390 (sample BIV9798) and OQ564391 (sample BIV6631). The remaining information was uploaded to figshare (https://figshare.com/s/cc8afa67637d2189e1ae). In detail, the files uploaded to figshare include the final unmasked and masked genome assemblies (Upi_v4.fa.gz and Upi_SM_v4.fa.gz), the annotation file (Upi_annotation_v4.gff3), predicted genes (Upi_genes_v4.fasta), predicted messenger RNA (Upi_mrna_v4.fasta), predicted open reading frames (Upi_cds_v4.fasta), predicted proteins (Upi_proteins_v4.fasta), as well as full table reports for Braker gene predictions and InterProScan functional annotations (Upi_InterPro_report_v4.txt.gz) and RepeatMasker predictions (Upi_RepeatMasker_v4.tbl.gz).
