# Supplementary material for: A PacBio Hi-Fi Genome Assembly of the Painter’s Mussel Unio pictorum (Linnaeus, 1758)
Source: Genome Biol Evol. 2023 Jun 21;15(7):evad116. doi: 10.1093/gbe/evad116 (PMC10329264; doi:10.1093/gbe/evad116)
Supplement: evad116_Supplementary_Data [file evad116_supplementary_data.zip › Table_S1.docx]

Table S2 - *Unio pictorum* Hifiasm genome assemblies’ tests general statistics.

|  |  | Hifiasm -s 0.75 (default) p_ctg | Hifiasm -s 0.55 p_ctg | Hifiasm -s 0.50 p_ctg | | Hifiasm -s 0.45 p_ctg | | Hifiasm -s 0.35 p_ctg |
| --- | --- | --- | --- | --- | --- | --- | --- | --- |
| Total number of Sequences (>= 1,000 bp) |  | 1,414 | 1,239 | 1,237 | | 1,169 | | 1,149 |
| Total number of Sequences (>= 10,000 bp) |  | 1,414 | 1,238 | 1,236 | | 1,169 | | 1,148 |
| Total number of Sequences (>= 25,000 bp) |  | 1,270 | 1,097 | 1,101 | | 1,035 | | 1,009 |
| Total number of Sequences (>= 50,000 bp) |  | 923 | 798 | 790 | | 752 | | 741 |
| Total length (>= 1,000 bp) |  | 2,741,830,755 | 2,647,790,403 | 2,631,946,495 | | 2,614,300,081 | | 2,603,599,464 |
| Total length (>= 10,000 bp) |  | 2,741,830,755 | 2,647,781,242 | 2,631,937,334 | | 2,614,300,081 | | 2,603,590,846 |
| Total length (>= 25,000 bp) |  | 2,738,984,137 | 2,644,973,785 | 2,629,252,637 | | 2,611,600,143 | | 2,600,812,966 |
| Total length (>= 50,000 bp) |  | 2,726,923,797 | 2,634,611,449 | 2,618,475,006 | | 2,601,793,057 | | 2,591,552,229 |
| N50 length (bp) |  | 9,091,481 | 9,428,870 | 9,952,985 | | 10,049,498 | | 10,868,501 |
| L50 |  | 86 | 81 | 78 | | 74 | | 74 |
| Largest contig (bp) |  | 44,859,553 | 44,859,553 | 44,859,553 | | 45,117,980 | | 44,859,553 |
| GC content, % |  | 34.83 | 34.83 | 34.83 | | 34.83 | | 34.83 |
| Total BUSCO for the genome assembly (%) |  |  |  | |  | |  |  |
| # Euk database |  | C:99.2% [S:94.9%, D:4.3%], F:0.8% | C:99.2% [S:94.5%, D:4.7%], F:0.8% | C:99.2% [S:94.5%, D:4.7%], F:0.8%, M:0.0% | | C:99.2% [S:93.7%, D:5.5%], F:0.8%, M:0.0% | | C:99.2% [S:94.1%, D:5.1%], F:0.8%, M:0.0% |
| # Met database |  | C:96.5% [S:92.9%, D:3.6%], F:2.4% | C:96.6% [S:92.9%, D:3.7%], F:2.3% | C:96.6% [S:93.0%, D:3.6%], F:2.3% | | C:96.6% [S:92.7%, D:3.9%], F:2.3% | | C:96.6% [S:92.9%, D:3.7%], F:2.3% |

# Euk: From a total of 303 genes of Eukaryota library profile.

# Met: From a total of 978 genes of Metazoa library profile.

#, + C: Complete; S: Single; D: Duplicated; F: Fragmented.
