# Supplementary material for: A PacBio Hi-Fi Genome Assembly of the Painter’s Mussel Unio pictorum (Linnaeus, 1758)
Source: Genome Biol Evol. 2023 Jun 21;15(7):evad116. doi: 10.1093/gbe/evad116 (PMC10329264; doi:10.1093/gbe/evad116)
Supplement: evad116_Supplementary_Data [file evad116_supplementary_data.zip › Table_S2.docx]

Table S2 - General statistics of the *Unio pictorum* final genome assembly (p_ctg); *Unio pictorum* alternative haplotypes genome assemblies (hap1 and hap2); other published freshwater mussel genome assemblies.

|  |  | Hifiasm -s 0.75 purge_dups p_ctg | Hifiasm -s 0.75 hap1 | Hifiasm -s 0.75 hap2 | | *Megalonaias nervosa* | | *Potamilus streckersoni* | | *Margaritifera margaritifera* V1 | | *Margaritifera margaritifera* V2 | *Unio delphinus* | | *Venustaconcha ellipsiformis* | | *Hyriopsis cumingii* | |
| --- | --- | --- | --- | --- | --- | --- | --- | --- | --- | --- | --- | --- | --- | --- | --- | --- | --- | --- |
| Total number of Sequences (>0 bp) |  | 670 | 3,357 | 2,702 | | 96,310 | | 2,366 | | 105,185 | | 1,700 | 1,254 | | 371,427 | | 77,26 | |
| Total number of Sequences (>= 10,000 bp) |  | 670 | 3,346 | 2,698 | | 54,764 | | 2,162 | | 15,384 | | 1,700 | 1,247 | | 26,952 | | 61,66 | |
| Total number of Sequences (>= 25,000 bp) |  | 643 | 2,602 | 2,374 | | 29,042 | | 1,831 | | 11,583 | | 1,202 | 968 | | 5,073 | | 35,57 | |
| Total number of Sequences (>= 50,000 bp) |  | 527 | 1,768 | 1,812 | | 12,699 | | 1,641 | | 9,265 | | 1,570 | 612 | | 1,456 | | 22,79 | |
| Total length (>= 0 bp) |  | 2,434,378,075 | 2,442,570,759 | 2,353,471,786 | | 2,361,438,834 | | 1,776,751,942 | | 2,472,078,101 | | 2,453,571,776 | 2,505,989,517 | | 1,590,012,607 | | 3,387,447,394 | |
| Total length (>= 10,000 bp) |  | 2,434,378,075 | 2,442,467,693 | 2,353,438,138 | | 2,193,448,794 | | 1,775,453,721 | | 2,293,496,118 | | 2,453,571,776 | 2,505,937,610 | | 541,544,602 | | 3,375,427,716 | |
| Total length (>= 25,000 bp) |  | 2,433,853,316 | 2,428,564,723 | 2,347,165,390 | | 1,768,523,103 | | 1,769,874,087 | | 2,236,013,546 | | 2,453,253,878 | 2,500,313,574 | | 231,252,884 | | 3,333,408,808 | |
| Total length (>= 50,000 bp) |  | 2,429,734,077 | 2,400,581,863 | 2,327,689,108 | | 1,194,323,847 | | 1,763,052,140 | | 2,152,307,394 | | 2,448,812,075 | 2,488,550,340 | | 107,178,666 | | 3,289,383,449 | |
| N50 length (bp) |  | 10,612,599 | 3,596,731 | 3,664,949 | | 50,662 | | 2,051,244 | | 288,726 | | 3,425,502 | 10,919,244 | | 6,657 | | 84,386,632 | |
| L50 |  | 71 | 181 | 174 | | 12,463 | | 245 | | 2,393 | | 207 | 67 | | 58,531 | | 15 | |
| Largest contig (bp) |  | 44,859,553 | 26,903,451 | 20628314 | | 588,638 | | 10,787,299 | | 2,510,869 | | 23,800,146 | 43,585,313 | | 313,274 | | 158,342,782 | |
| GC content, % |  | 34.82 | 34.84 | 2,698 | | 35.82 | | 33.79 | | 35.42 | | 35.30 | 35.07 | | 34.19 | | 36.07 | |
| Clean Paired-End (PE) Reads Alignment Stats |  |  |  | |  | |  | |  | |  | | |  | |  | |  |
| Percentage of Mapped WGS PE (%) |  | 99.49 | - | - | | - | | - | | - | | - | - | | - | |  | |
| Percentage of Mapped WGS PacBio (average per cell %) |  | 99. 51 | - | - | | - | | - | | - | |  |  | |  | |  | |
| Percentage of Mapped RNA-seq PE (%) |  | 94.83 | - | - | | - | | - | | - | | - | - | | - | |  | |
| Total BUSCO for the genome assembly (%) |  |  |  | |  | |  | |  | |  | | |  | |  | |  |
| # Euk database |  | C:99.2% [S:96.1%, D:3.1%], F:0.8% | C:95.2% [S:92.5%, D:2.7%], F:2.0%, | C:94.1% [S:91.4%, D:2.7%], F:2.0% | | C:70.6% [S:70.2%, D:0.4%], F:14.9% | | C:98.1% [S:97.3%, D:0.8%], F:0.8% | | C: 86.8% [S: 85.8%, D:1.0%], F: 5.9% | | C:99.2% [S:97.6%, D:1.6%], F:0.4% | C:98.5% [S:96.1%, D:2.4%], F:1.6% | | C:45.9% [S:45.5%, D:0.4%], F:36.9% | | C:93.0% [S:92.2%, D:0.8%], F:3.1% | |
| # Met database |  | C:96.3% [S:93.7%, D:2.6%], F:2.4% | C:92.3% [S:90.1%, D:2.2%], F:2.5% | C:92.9% [S:90.6%, D:2.3%], F:2.8% | | C:71.5% [S:70.1%, D:1.4%], F:14.5% | | C:95.0% [S:93.6%, D:1.4%], F:2.3% | | C: 84.9% (S: 83.8%, D: 1.1%), F: 4.9% | | C:96.9% [S:95.5%, D:1.4%], F:2.0% | C:96.5% [S:94.4%, D:2.1%], F:2.3% | | C:53.7% [S:52.8%, D:0.9%], F:29.7% | | C:93.6% [S:92.3%, D:1.3%], F:2.3% | |
| Masking Repetitive Regions and Gene Prediction |  |  |  | |  | |  | |  | |  | | |  | |  | |  |
| Percentage masked bases (%) |  | 49,98 | - | - | | 25.00 | | 51.03 | | 59.07 | | 57.32 | 52.83 | | 36.29 | | 50.86 | |
| Number of mRNA |  | 46,138 | - | - | | 49,149 | | 41,065 | | 40,544 | | 48,314 | 44,382 | | - | | 37,681 | |
| Protein coding genes (CDS) |  | 46,138 | - | - | | 49,149 | | 41,065 | | 35,119 | | 48,314 | 44,382 | | - | | 37,681 | |
| Functional annotated genes |  | 34,137 | - | - | | - | | - | | 31,584 | | 35,649 | 32,089 | | - | | - | |
| Total gene length (bp) |  | 832,204,995 | - | - | | - | | - | | 902,994,752 | | 1.13 Gb | 869,540,056 | | - | | - | |
| Total BUSCO for the predicted proteins (%) |  |  |  | |  | |  | |  | |  | | |  | |  | |  |
| + Euk database |  | C:96.1% [S:86.7%, D:9.4%], F:3.5% | - | - | | - | | - | | C:90.6% [S:81.2%, D:9.4%], F:3.9% | | C:97.6% [S:83.9%, D:13.7%], F:2.0% | C:96.8% [S:88.2%, D:8.6%], F:2.7% | | - | | - | |
| + Met database |  | C:97.4% [S:85.7%, D:11.7%], F:2.0% | - | - | | - | | - | | C:92.6% [S:82.3%, D:10.3%] , F:3.2% | | C:98.7% [S:84.7%, D:14.0%], F:0.8% | C:97.3% [S:86.0%, D:11.3%], F:2.3% | | - | | - | |

# Euk: From a total of 303 genes of Eukaryota library profile.

# Met: From a total of 978 genes of Metazoa library profile.

+ Euk: From a total of 255 genes of Eukaryota library profile.

+ Met: From a total of 954 genes of Metazoa library profile.

#, + C: Complete; S: Single; D: Duplicated; F: Fragmented.
