# Supplementary material for: A PacBio Hi-Fi Genome Assembly of the Painter’s Mussel Unio pictorum (Linnaeus, 1758)
Source: Genome Biol Evol. 2023 Jun 21;15(7):evad116. doi: 10.1093/gbe/evad116 (PMC10329264; doi:10.1093/gbe/evad116)
Supplement: evad116_Supplementary_Data [file evad116_supplementary_data.zip › Supplementary material legends.docx]

**Supplementary figure legend**

fig. S1 – A) Bioinformatics pipeline applied for the whole genome assembly and annotation. B) *Unio* *pictorum* genome assembly assessment using KAT comp tool to compare the PacBio HiFi k-mer content within the genome assembly before running purge_dups. Different colours represent the read k-mer frequency in the assembly.

**Supplementary tables legends**

Table S1 – *Unio pictorum* Hifiasm genome assemblies’ tests general statistics.

Table S2 – General statistics of the *Unio pictorum* final genome assembly (p_ctg); *Unio pictorum* alternative haplotypes genome assemblies (hap1 and hap2); other published freshwater mussels genome assemblies.
