# Supplementary figures and images for: A PacBio Hi-Fi Genome Assembly of the Painter’s Mussel Unio pictorum (Linnaeus, 1758)
Source: Genome Biol Evol. 2023 Jun 21;15(7):evad116. doi: 10.1093/gbe/evad116 (PMC10329264; doi:10.1093/gbe/evad116)

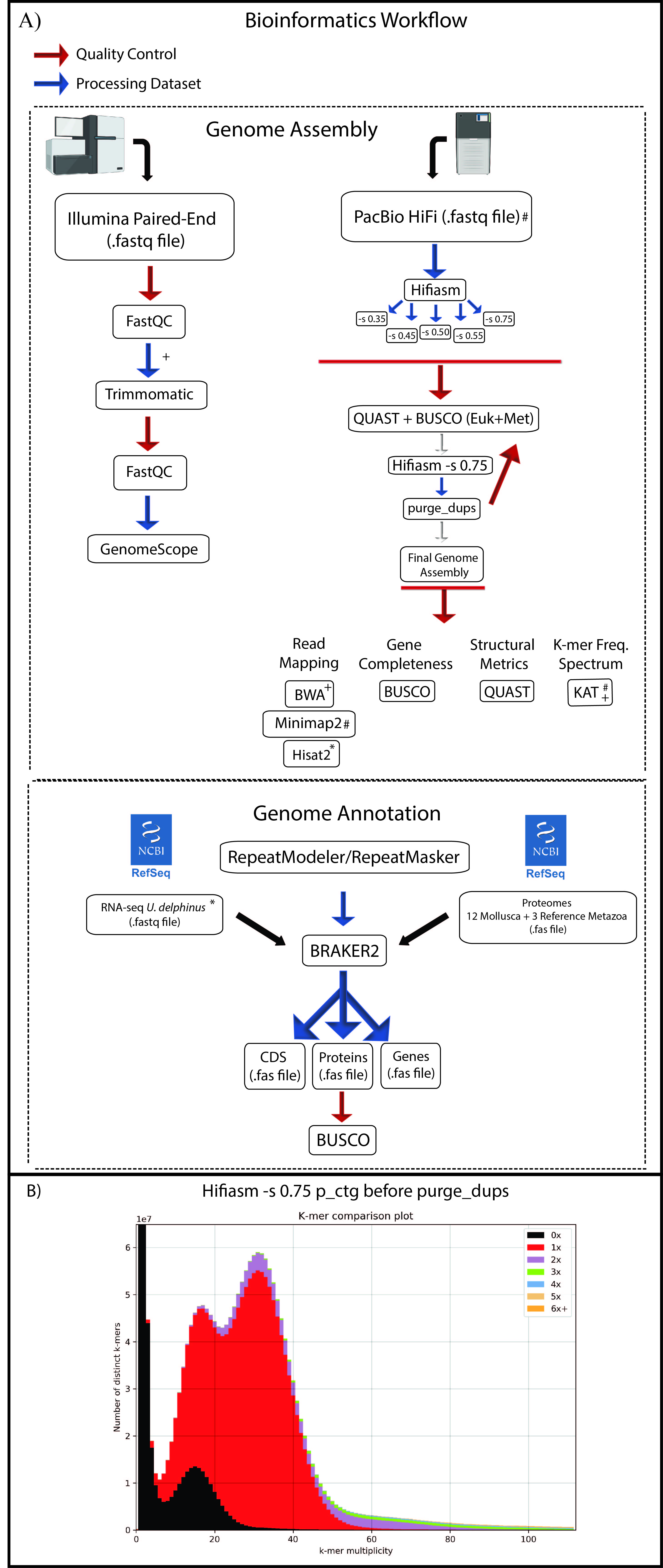

Supplement: evad116_Supplementary_Data [file evad116_supplementary_data.zip › fig.S1.jpg]
